# Assessing computational genomics skills: Our experience in the H3ABioNet African bioinformatics network

**DOI:** 10.1371/journal.pcbi.1005419

**Published:** 2017-06-01

**Authors:** C. Victor Jongeneel, Ovokeraye Achinike-Oduaran, Ezekiel Adebiyi, Marion Adebiyi, Seun Adeyemi, Bola Akanle, Shaun Aron, Efejiro Ashano, Hocine Bendou, Gerrit Botha, Emile Chimusa, Ananyo Choudhury, Ravikiran Donthu, Jenny Drnevich, Oluwadamila Falola, Christopher J. Fields, Scott Hazelhurst, Liesl Hendry, Itunuoluwa Isewon, Radhika S. Khetani, Judit Kumuthini, Magambo Phillip Kimuda, Lerato Magosi, Liudmila Sergeevna Mainzer, Suresh Maslamoney, Mamana Mbiyavanga, Ayton Meintjes, Danny Mugutso, Phelelani Mpangase, Richard Munthali, Victoria Nembaware, Andrew Ndhlovu, Trust Odia, Adaobi Okafor, Olaleye Oladipo, Sumir Panji, Venesa Pillay, Gloria Rendon, Dhriti Sengupta, Nicola Mulder

**Affiliations:** 1 High-Performance Biological Computing, Roy J. Carver Biotechnology Center, University of Illinois at Urbana-Champaign, Urbana, Illinois, United States of America; 2 Institute for Genomic Biology and National Center for Supercomputing Applications, University of Illinois at Urbana-Champaign, Urbana, Illinois, United States of America; 3 Sydney Brenner Institute for Molecular Bioscience, University of the Witwatersrand, Johannesburg, South Africa; 4 Department of Computer and Information Sciences, Covenant University, Ota, Nigeria; 5 Covenant University Bioinformatics Research (CUBRe), Covenant University, Ota, Nigeria; 6 Directorate, Center for System and Information Service, Covenant University, Ota, Nigeria; 7 National Biotechnology Development Agency (NABDA), Federal Ministry of Science and Technology (FMST), Abuja, Nigeria; 8 Computational Biology Division, Department of Integrative Biomedical Sciences, Institute of Infectious Disease and Molecular Medicine, University of Cape Town, South Africa; 9 Centre for Proteomic and Genomic Research, Cape Town, South Africa; 10 School of Electrical & Information Engineering, University of the Witwatersrand, Johannesburg, South Africa; 11 Research Unit in Bioinformatics (RUBi), Department of Biochemistry and Microbiology, Rhodes University, Grahamstown, South Africa; 12 Botswana Harvard AIDS Institute Partnership, Gaborone, Botswana; Ontario Institute for Cancer Research, CANADA

## Abstract

The H3ABioNet pan-African bioinformatics network, which is funded to support the Human Heredity and Health in Africa (H3Africa) program, has developed node-assessment exercises to gauge the ability of its participating research and service groups to analyze typical genome-wide datasets being generated by H3Africa research groups. We describe a framework for the assessment of computational genomics analysis skills, which includes standard operating procedures, training and test datasets, and a process for administering the exercise. We present the experiences of 3 research groups that have taken the exercise and the impact on their ability to manage complex projects. Finally, we discuss the reasons why many H3ABioNet nodes have declined so far to participate and potential strategies to encourage them to do so.

## Author summary

Many programs have been developed to boost the technical and computational skills of scientists working in low to medium income countries (LMIC), who often struggle to remain competitive with their peers in more developed parts of the world. Typically, these programs rely on intensive workshops where students acquire and exercise these skills under the supervision of experienced trainers. However, when trainees return to their home institutions, even after extensive exposure to state of the art techniques, they often find it difficult to put the skills they have acquired into practice and to establish themselves as fully independent practitioners. We have attempted to build a framework through which teams of scientists in African research groups can demonstrate that they have acquired the necessary skills to analyze different types of genomic datasets. Three teams of scientists who have successfully submitted to this assessment exercise report their positive experiences. Many potential participants have so far declined the opportunity, and we discuss the reasons for their reluctance as well as possible ways to facilitate their engagement and provide them with incentives. We argue that assessments such as this could be part of any program aiming to develop technical skills in scientists wishing to support genomic research programs.

This is a *PLOS Computational Biology* Education paper.

## Introduction

The Human Heredity and Health in Africa (H3Africa) initiative [1] (http://h3africa.org/), which is funded jointly by the National Institutes of Health (NIH) in the United States and the Wellcome Trust in the United Kingdom, aims to bring genomic approaches to the study of the genetic and environmental determinants of disease susceptibility on the African continent. To meet this primary goal, H3Africa funds multiple research projects led by African scientists that collect genome-wide data as well as extensive phenotypic information on study participants. Some projects are case-control studies; others are following cross-sectional cohorts. To the extent possible, the collection of genotypic and phenotypic data across research projects has been harmonized, and data-sharing agreements have been put in place so that data can be aggregated and analyzed across target diseases and participant populations. Other priorities of H3Africa are to develop the necessary expertise among African scientists to collect and analyze genome-wide datasets and to help establish networks of scientists who can collaboratively bring complex research projects to fruition.

The H3Africa Bioinformatics Network, H3ABioNet [2] (http://h3abionet.org/), was funded by NIH to support the H3Africa program and to build capacity on the continent for managing and analyzing large-scale genomic and biomedical data. H3ABioNet comprises 32 African research groups (“nodes”) in 14 countries, plus affiliates in the US and the UK. It has multiple missions spanning infrastructure building, data management and curation, training, and capacity development for H3Africa researchers, networking (in both the technical and social meanings of the term), setting standards for data collection, and generally providing the computational tools and services required for the success of H3Africa research projects. Less explicitly, but just as importantly, H3ABioNet hopes to free African scientists from their dependence on collaborators in developed countries for the analysis of data collected on the African continent, which is fraught with issues of data ownership, attribution of credit in scientific publications, and the ability to present the results in their cultural and political context.

When H3Africa research projects were conceived and funded, the majority relied on collaborators in developed countries for much of the data generation (sequencing, genotyping) and analysis. Under H3Africa, Africans are the principal investigators (PIs) and control the research budgets and logistics, but the pattern of using African scientists primarily for accruing study participants and collecting samples and leaving the “science” to others has continued. An obvious reason for outsourcing is the dearth of groups on the African continent with the necessary infrastructure and skills to manage and analyze the large volumes of data being generated by the research projects [3]. In addition, many of the PIs have existing collaborations with scientists outside Africa whom they trust more than potential collaborators on the continent whom they do not know and who may not have a well-established record of accomplishment. This leads to a scarcity in novel datasets to work on, which perpetuates the cycle.

During the first general assembly of H3ABioNet in November 2012, a small group of scientists gathered to discuss how the network’s affiliated nodes could establish themselves as credible collaborators able to manage and analyze genomic datasets for H3Africa projects and beyond. Everyone agreed that this should be one of the missions of the network. The discussion was lively and touched upon topics such as the diversity of participating nodes and corresponding expectations, whether the capabilities of individual nodes should be monitored and assessed on a regular basis, what core skills should be measured, who would be mandated to carry out the assessments, and whether the assessments would be voluntary or mandatory. The possibility of including H3Africa research groups in the evaluation process was brought up, but because they are not funded by the H3ABioNet grant, this idea was not pursued further. What emerged was a Node Assessment Task Force (NATF), whose mandate was to draft its own “Terms of Reference” for approval by the general assembly and to work out an assessment protocol specifying the modalities and procedures used to assess the ability of a node to analyze specific types of data. This report examines how this was done, documents the experiences of some of the nodes that underwent the assessment, and reflects on the challenges encountered in measuring research capacity in the African context.

## The node-assessment exercise

### Scope of the exercise

The first decision the NATF had to make was which specific skills should be assessed in participating nodes. Given the emphasis of many H3Africa projects on discovering and measuring variation in the genomes of African populations, we initially focused on 2 techniques: (1) the calling of variants (single nucleotide polymorphisms [SNPs] and small indels) in individual study participants from whole genome or whole exome high-throughput sequence data and (2) the processing of raw data from genotyping chips to call variants in a patient population and subsequent genome-wide association studies (GWAS) of genotypes obtained from case and control populations to identify variants potentially associated with a disease phenotype. Subsequently, scientists from some of the H3ABioNet nodes suggested that the NATF also consider techniques in wider use at African institutions, with which they were more likely to be confronted. Thus, the NATF added (3) the analysis of RNA-seq data, from read processing and alignment to a reference genome to the identification of differentially expressed genes across samples and (4) the identification of Operational Taxonomic Units (OTUs) from selectively amplified 16S ribosomal DNA sequences derived from microbial populations and the comparison of OTU abundances within and across samples. These 4 techniques are typical of the types of data-analysis challenges encountered by bioinformatics core facilities around the world, and our expectation was that most H3ABioNet nodes should be able to demonstrate competence in at least 1 of them.

To ensure that all candidate nodes would have proper guidance on how to perform the chosen analyses, the NATF produced Standard Operating Procedures (SOPs) for all of them, outlining the necessary steps, suggesting software that would be most appropriate, and pointing out pitfalls along the way. These SOPs were posted to the H3ABioNet website, where they are publicly available (see http://h3abionet.org/tools-and-resources/sops).

### Implementation

For each of the 4 exercises, the NATF prepared 2 types of datasets. A small practice dataset was posted on the H3ABioNet website to allow the team to prepare for the actual exercise without using excessive computational resources and within a short turnaround time. A set of test datasets, more realistic in size but still easily downloadable, was also prepared to be used for the actual exercise. The test datasets were anonymized to the extent possible to avoid having the candidates reproduce analyses from the literature. An effort was also made to ensure that datasets were comparable to each other to avoid favoring one candidate node over another. For the whole genome sequencing (WGS)–variant calling exercise, we produced synthetic data that contained variants typical of African populations using a read simulator developed at the University of Illinois, NEAT (https://github.com/zstephens/neat-genreads). This ensured that the set of variants to be discovered was known in advance and was thus verifiable. Similarly, the genotypes used as input for the GWAS analyses were simulated to be derived from an admixed population, with a limited number of variants contributing significantly to the observed phenotype. For the SNP—chip genotype calling, raw intensity files from a real microarray experiment were used, with a unique subset being selected for each node, to maintain uniqueness. For the other exercises, we used subsets of data from large published studies.

We envisioned that taking 1 of the assessment exercises would be a 3-step process: (1) preparation of the team, initially by participating in workshops organized by H3ABioNet or other training organizations, followed by setting up the necessary software and running through the SOP using the practice datasets; (2) announcing readiness to start and receiving the test datasets, followed by 6 weeks of computational analysis and writing of the report; (3) evaluation of the report by a panel of external experts. In practice, this worked quite well. Experiences of individual nodes with the process are summarized in the next section. The 6-week deadline between the release of the test datasets and the delivery of the report turned out to be a reasonable compromise between leaving enough time for a thorough analysis and not letting the project linger for too long.

Since the inception of the assessment exercise in late 2013 until the time of this writing (November 2016), 3 nodes have taken the plunge. The Bioinformatics group at the University of the Witwatersrand (Wits; Professor Scott Hazelhurst) and the Covenant University Bioinformatics Research group (CUBRe; Professor Ezekiel Adebiyi) took the genotyping and GWAS analysis assessment. The Computational Biology group at the University of Cape Town (CBIO; Professor Nicola Mulder) took the variant calling from WGS data assessment. Their experiences are described in the next section.

## Experiences from the nodes

### Wits—University of the Witwatersrand, South Africa

The Wits H3ABioNet node undertook the accreditation process in GWAS in April/May 2014. Mastering this workflow is one of the key priorities in supporting the H3Africa AWI-Gen project (https://www.wits.ac.za/research/sbimb/research/awi-gen/), which aims to gain an understanding of and response to the interplay between genetic, epigenetic, and environmental risk factors for obesity and associated cardiometabolic disease in sub-Saharan Africa. The team was cognizant of the work and time that this exercise would take, given existing work commitments. Therefore, they decided to use the exercise as a learning opportunity as much as possible. First, they organized and presented a training workshop from 14–17 April 2014, partially supported by H3ABioNet. Second, they hosted an H3ABioNet intern, Ms. Lerato Magosi, from the Botswana Harvard AIDS Institute Partnership, during the preparation and execution of the exercise. Third, they organized a “shadow” team of 6, comprising postgraduate students and a postdoctoral fellow, who performed many of the analyses in parallel with the core team of 5.

The whole exercise was a valuable one for the node. Coupling it with a training program was a sensible approach, since they could use the experience as a focus for a set of activities that got the whole team involved. The internship was very successful, as the intern contributed significantly to the work of the core team and to the content of the report and left with a solid core competency in the analysis of GWAS data.

The shadow team helped with some of the analyses, so the benefit of the experience spread beyond the core team. Most of the shadow team had already received some formal training in GWAS, so this was an opportunity to consolidate their experiences. To participate in the shadow team, members were asked to commit 16 hours over a 4-week period (above any training time). The shadow and core teams had joint formal weekly meetings, as well as significant informal contact. Mostly, the shadow team replicated what the core team did, but in a few cases, they used different tools or ran them with different parameters. The shadow team compared their results with those of the core team, received feedback, and discussed their results and plans. The work of the shadow team supported the core team by increasing the confidence in the robustness of the results and saving the core team time. Two members of the shadow team have gone on to lead the bioinformatics analysis of the AWI-Gen pilot GWAS study.

Besides improving the capacity of the group, the exercise showed the importance of developing SOPs and building pipelines for analysis. In a follow-up internship, the intern developed a prototype pipeline for GWAS. This has fed into a much larger pipelines project currently underway in H3ABioNet.

### CBIO—University of Cape Town, South Africa

The CBIO team at the University of Cape Town (UCT) took the WGS—variant calling assessment exercise in October/November 2015. At the time, it was already actively engaged in setting up variant-calling workflows for the H3Africa genotyping chip design project, one of whose goals was to discover variants in WGS data from over 3,000 African individuals. Therefore, taking the exercise was an excellent opportunity to hone the necessary skills and test the workflows developed for the chip design project. Like Wits, the CBIO group organized itself into a core team and a smaller shadow team working in parallel, with the shadow team focused primarily on skills development but also free to try alternative approaches. The CBIO shadow team initially concentrated on providing literature reviews of the various tools available to gain familiarity with them and with the data and trends in the field, and to strengthen their ability to interpret the results. The shadow team also worked on gaining a better understanding of various variant annotation tools such as CliniVar and Variant Effect Predictor (VEP) from Ensembl. The CBIO shadow team tried alternative tools and parameters to the recommended SOP (e.g., using Novoalign instead of BWA-MEM) and benchmarked the wall times of these different applications of a variety of high-performance computing (HPC) environments available to the CBIO group. These included the newly commissioned CBIO cluster and the UCT central HPC, and the results helped provide invaluable documentation for future projects based on the available hardware. This approach enabled shadow team members to get a better understanding of the steps involved in WGS—variant calling (theoretical and practical), help set up and execute some of the nonstandard tools they had available to them, and discuss and compare results with the core team. This has led to a better understanding of the WGS—variant calling pipeline, and one of the shadow team members (Hocine Bendou) was instrumental in the creation of Common Workflow Language bindings and Docker images for the WGS—variant calling pipeline for a subsequent H3ABioNet project. CBIO also hosted an intern from another node, Magambo Phillip Kimuda, who is part of the H3Africa TrypanoGEN project (http://www.trypanogen.net/) at Makerere University in Uganda and was at the time enrolled in the Bioinformatics MSc program at Rhodes University. Together, they produced an exhaustive report of their work, with a set of supplementary documents reporting details of the various steps in the assessment.

Besides the next-generation sequencing (NGS) data-analysis skills gained, the exercise provided the group with an ideal opportunity to learn teamwork and manage a collaborative project. They developed skills and lessons to effectively

- work in a team with members from different technical backgrounds and
- collaborate on a project where most members have other priorities, commitments, and time constraints.

From a technical point of view, the team learned to use new tools and strategies for the first time during this exercise: Slack (https://slack.com) is an online collaboration tool favored by software development teams. Initially, the CBIO team was using emails, conferencing tools such as Skype, and alert notifications enabled by BitBucket, but given the complexity of the project, the unsynchronized times that members could work on the assessment, and the fragility of email chains, a decision was made to try new collaborative tools currently used by other teams. The experience was very much like that of Internet Relay Chat (IRC) rooms, and Slack has integration with other tools they were using (Google Docs, Bitbucket). As hoped, it turned out to be powerful as a central place for discussion and updates on what people had accomplished and were working on, enabling CBIO members to quickly catch up and determine what parts of the assessment they needed to work on without having to trawl through emails and multiple versioned documents. This also prevented duplication of work by CBIO members, as input, output and processing files, and scripts were readily locatable, and comparisons between results from various tools and parameter runs could be easily made. The ability to obtain a real-time response within the context of any question or update being posted for the assessment was also found to be extremely beneficial. The use of these collaborative tools was supplemented with regular weekly planning meetings. A Google Drive folder was used as the central repository for their documentation, and BitBucket was used as the central repository for code and as a ticketing system to track tasks. Using a distributed version control system meant that different members could simultaneously work on parts of the codebase. These collaborative tools are currently in use by the CBIO team and have been adopted as a standard practice for most of the collaborative projects they conduct. In summary, the assessment exercise provided an incentive for the group to hone its collaborative project-management and technical skills and to reinforce and demonstrate its NGS data-analysis skills.

### CUBRe—Covenant University, Nigeria

The CUBRe group undertook the GWAS accreditation process in October/November 2015. While the group’s core strengths are in biological network reconstruction and modeling, it decided that acquiring a strong competence in GWAS data analysis was essential to its participation in the H3Africa research program and to establishing its credibility as a strong collaborator in computational genomics.

To prepare itself, the group took advantage of several opportunities. Firstly, the team carefully studied the documentation of the software used for the GWAS—analysis workflows as presented on the H3ABioNet accreditation website. From this information, they updated and tested the software installed on their HPC infrastructure. Secondly, CUBRe organized a GWAS—analysis workshop at Covenant University in the summer of 2015, where veterans of the 2014 Wits exercise (Scott Hazelhurst and Shaun Aron) came to share their experience and expertise. Interestingly, one of CUBRe’s recent graduates, Segun Fatumo, had joined the group of Manjinder Sandhu at the Wellcome Trust Sanger Institute as a postdoctoral fellow. There, he was working on the large-scale genetic analysis of cardiometabolic traits in African populations and applying advanced GWAS—analysis methods in his research. Segun Fatumo was also a trainer at this workshop, which was attended by most of the members of the CUBRe accreditation team. Because of last-minute scheduling problems, the last part of the workshop had to be canceled. Ezekiel Adebiyi, the leader of the CUBRe group, gave an overview of the remaining topics and assigned exercises from published papers so that the results from the analyses could be compared to those obtained by the authors.

Well-prepared by the workshop and knowing it could count on advice from its trainers, the team was able to work through the practice data and declare its readiness in October. It did a professional job at the data-processing and analysis stages of the exercise and augmented its investigations by reporting on the potential functional significance of the variants found to be statistically associated with the selected phenotype. For the latter, it leveraged its expertise in biological network analysis.

## Lessons learned

The node-assessment exercises described above were designed to test a set of technical competencies of the type that a core facility attached to a research institution should be able to demonstrate. These technical skills are among those that the H3ABioNet training activities are aiming to foster in its participating nodes as well as in H3Africa research groups. We are fully aware that these are only some of the competencies sought by African researchers in their prospective collaborators and that the ability to design projects from a biomedical, statistical, or computational perspective is not being addressed by the exercises. This ability can only be honed through a combination of formal training, research experiences in top-level groups, and continued involvement in complex projects. However, it is also clear that there is an urgent need to develop and rigorously assess the types of analytical skills tested here, without which projects using genome-level data collection and analysis methods cannot succeed, and which are essential to the development of a broader level of scientific excellence.

The design and implementation of the exercises were a clear success from an operational standpoint in that the datasets, the workflows described in the SOPs, and the timelines assigned for completing the exercises were realistic and resulted in solid reports. Participation in the exercises was beneficial to the nodes and helped them support the research projects they were engaged in. Feedback from the nodes that took the exercise was very positive and constructive and led to improvements and fixes in the SOPs and in the datasets, which will enhance the experience for the next candidates. External experts noted that the submitted reports were of uniformly high quality. Each of the participating nodes was able to assemble a team that worked cohesively on the analyses and the writing of the reports.

From the perspective of the NATF, the amount of work required to set up and administer the exercises turned out not to be trivial. Drafting detailed SOPs, identifying or generating appropriate datasets, testing them with standard workflows, preparing “correct” answers to the analyses against which the participants’ efforts could be tested, and managing the distribution of the datasets are all labor-intensive steps. Furthermore, finding external experts able and willing to read and rate the reports and keeping them engaged was also more difficult than we had imagined. Anyone planning to implement an assessment such as the one we conducted must be fully cognizant of the necessary investments in staff time and expertise.

On the other hand, the level of participation was extremely disappointing. Of the 32 African nodes in H3ABioNet, only 3 submitted candidacies, of which, 2 were from South Africa, a country with a high level of scientific development. Two of the 4 exercises have yet to find takers. While H3ABioNet offered plentiful opportunities for African scientists to learn the skills required to participate, only a single node outside South Africa took up the challenge within the first 3 years. This raises 2 important questions: (1) Why did most of the nodes choose not to participate? (2) What could H3ABioNet or, for that matter, any organization promoting capacity development in Africa do to encourage scientists to seize an opportunity to showcase their capabilities and show that their training had paid off?

We can provide a partial answer to the first question from comments made by H3ABioNet node managers explaining their decision not to participate or to delay participation. The most frequently heard reason was that the amount of time and effort (staff and student hours) required to prepare and participate was too high, that participation would not lead to publishable results, and that obligations to their other funders precluded the necessary investments. The second reason was that the technical skills required to take the exercises were outside the scientific focus of the node, and that becoming candidates would require rethinking the research directions of the node and training its staff in noncore research skills. A third reason was that the node did not have the level of staffing that would allow them to even consider participating. We believe that a fourth reason, never publicly voiced, but nevertheless very real, was the fear of failure, of not being able to measure up to the standards set by the NATF or produce a report that would be sufficiently knowledgeable and detailed. The lack of self-confidence of many African scientists has been a factor in the asymmetry of their relationships with first world collaborators and, in our opinion, is one of the issues to be addressed in capacity-development efforts.

Regarding the second question, encouraging the nodes to participate in the exercise has been one of the major goals of H3ABioNet over the last 2 years. The network would be considered a partial failure if its participating groups could not demonstrate a level of technical and scientific competence that would make them attractive collaborators for H3Africa research projects. Three specific measures were taken to make taking the exercise a more attractive option. Firstly, participation of node scientists in H3ABioNet training events, or the awarding of internships in institutions abroad, was made contingent on a commitment to participate in an assessment exercise that applies the skills learned during the training. Secondly, this participation has become part of the contractual obligations of the node, and failure to comply could result in the suspension of payments from the NIH grant. To accompany these rather coercive measures, H3ABioNet is making available additional training opportunities and materials, and the candidate nodes are encouraged to seek the help of more experienced nodes during the preparation phase, as exemplified by the CUBRe group being helped by the Wits group. We are hoping that this combined approach will result in a significantly increased rate of participation, and, indeed, a significant number of nodes have announced that they were getting ready to take the exercise in late 2016 or the first half of 2017. We also considered narrowing the scope of some of the more complex exercises (e.g., the GWAS analysis or variant calling) by subdividing them into smaller tasks, but we decided against this approach because it would no longer provide realistic challenges for the candidate nodes.

It is also worth noting that a node taking an assessment exercise has the possibility to familiarize scientists from other nodes with the process, thus helping them prepare to take it themselves (e.g., Lerato Magosi, from Botswana at Wits, or Phillip Kimunda, from Uganda at CBIO). This is one of the benefits of being part of a larger network and helps spread skills across the network. More directly, we found that it led to new collaborative relationships between the participating members. We hope that it will become a standard approach as more and more nodes participate.

## Conclusions

We have designed and implemented a framework for assessing the capacity of scientific research groups, primarily from scientifically less advanced countries, to perform specific types of widely used analyses of high-throughput genomic data. This framework has been field-tested and has proven effective in producing a fair assessment of the candidate groups. It could be implemented more widely in the framework of capacity-building programs worldwide and as a quality check on groups providing core services for genomic research. Specifically, the approach combining SOPs to guide the candidates’ efforts, practice datasets to let them test and improve their workflows, anonymized and in some cases synthetic test datasets for a fair assessment of their skills, and an evaluation by external experts could be generalized to almost any assessment of data analysis skills. We would be happy to share both the relevant materials and our experiences.

Too many training programs deployed in LMIC concentrate on the delivery of scientific knowledge and technical skills but neglect to test whether the trainees, when returning to their home institutions, can use these skills in real-life situations. The reasons for not being able to do so are many, including insufficient (1) access to a working computational infrastructure, (2) support from their hierarchy, (3) motivation from their institutional environment, (4) help from colleagues, or (5) access to scientifically relevant datasets. The approach that we propose was not designed to address institutional issues, but the ability to get recognition for the host institution may act as a motivating factor. However, in our opinion, the possibility of including a longer-term, realistic assessment of the acquisition of technical and scientific skills undertaken in the home institution of the trainees is an option to consider for most, if not all, capacity-building programs.

As evidenced from the poor initial uptake of our program within the H3ABioNet network, there is bound to be significant pushback from both researchers and their host institutions because of the potential drain on their resources or because of concerns about their ability to deliver. Engaging the researchers and their institutions early, making sure that they can be properly rewarded for demonstrating their enhanced capabilities, and ensuring that they understand the benefits of doing so will be key to a successful implementation.
